# The efficacy and safety of endoscopy-assisted anterior cervical discectomy and fusion for the treatment of cervical spondylotic myelopathy

**DOI:** 10.3389/fsurg.2026.1700982

**Published:** 2026-01-26

**Authors:** Jingchao Wei, Shangju Gao, Xiaohua Li, Yusong Guo, Yuxin Meng, Wenyi Li

**Affiliations:** Department of Orthopedics Surgery, Hebei General Hospital, Shijiazhuang, Hebei, China

**Keywords:** anterior cervical discectomy andfusion, cervical spondylotic myelopathy, efficacy, endoscopy, minimally invasive

## Abstract

**Objective:**

The aim of this study was to describe the surgical technique of endoscopy-assisted anterior cervical discectomy and fusion (ACDF), to evaluate the advantages, efficacy, and safety of this procedure for the treatment of cervical spondylotic myelopathy (CSM).

**Methods:**

The clinical data of patients with CSM treated with endoscopy-assisted ACDF from January 2023 to December 2023 were retrospectively reviewed. And 35 patients, including 13 females and 22 males, were included in this study. Endoscopic assisted ACDF surgery was described step by step in detail, and clinical and imageological assessment were performed before and after operation and follow-up.

**Results:**

All 35 patients underwent endoscopy-assisted ACDF surgery successfully, and were followed up for 12.9 ± 2.1 months (range 9∼18 months). The operation time was 74.4 ± 10.7 min (range 60∼100 min). Postoperative drainage volume was 14.1 ± 5.8 mL (range 5∼25 mL). No complications were observed. There were no complications, aggravation of neurological symptoms after operation, and the JOA score at the last follow-up was significantly improved compared with that before operation (15.7 ± 0.8 vs. 10.3 ± 1.9, *P* < 0.001). At the last follow-up, the C2-C7 Cobb angle was significantly higher than that before operation (*P* < 0.001), and *Δ* Cobb angle was 7.4 ± 2.5˚, and all patients achieved bony fusion.

**Conclusions:**

Endoscopy-assisted ACDF, which combined the uniaxial spinal endoscopy with traditional ACDF, achieved satisfactory short-term clinical efficacy and safety in the treatment of CSM.

## 1 Introduction

Anterior cervical discectomy and fusion (ACDF) is a classic surgical procedure for the treatment of cervical spondylotic myelopathy (CSM). It can directly decompress the ventral side of the spinal cord and restore the physiological cervical curvature through the natural anatomical gap approach, and has the advantages of less trauma, less bleeding and faster recovery (1). However, due to the limitations of eyesight visual acuity, there are deficiencies in surgical field of view, precision, and the ability to recognize neural and vascular tissues, which increases the risk of spinal cord injury (2).

In recent years, microscopy-assisted ACDF has gradually been adopted in the treatment of cervical spondylosis. This technique integrates traditional ACDF with microscopy technology, utilizing the microscopic magnification capabilities to compensate for the shortcomings of traditional ACDF to some extent. However, it still remains the “hand-eye dissociation” disadvantage, which is one of the unsolved challenges of microscope operation. Surprisingly, depending on the widespread application of endoscopic technology, the problem is expected to be solved.

Endoscopy-assisted ACDF was innovated to promote minimally invasive and accelerate recovery. The aim of this study was to describe the surgical technique of endoscopy-assisted ACDF, and to evaluate the advantages, efficacy, and safety of this procedure for the treatment of CSM.

## 2 Material and methods

### 2.1 Study population

This retrospective study was approved by Hebei General Hospital Ethics Committee (No. 2025-LW-0117). The clinical data of patients with CSM treated with endoscopy-assisted ACDF in Hebei General Hospital from January 2023 to December 2023 were retrospectively reviewed.

The inclusion criteria were as follows: (1) compression on the ventral side of the cervical spinal cord observed on MRI, which was consistent with the symptoms and signs; (2) endoscopy-assisted ACDF surgery performed; (3) complete imagological data, including cervical spine x-ray, CT and MRI examination before operation; and (4) complete clinical and follow-up data. The exclusion criteria were as follows: (1) combined with ossification of the posterior longitudinal ligament or hypertrophy of the ligamentum flavum; (2) combined with cervical spine deformity, tumor or infection; and (3) patients with previous cervical spine surgery.

A total of 35 patients with CSM were included in this study, including 13 females and 22 males, with mean age of 56.5 ± 10.1 years (range 35∼72 years). All patients were followed up for more than 9 months. All patients received surgical treatment by single-level endoscopy-assisted ACDF surgery, including 3 cases in C3-4, 7 cases in C4-5, 22 cases in C5-6, and 3 cases in C6-7.

### 2.2 Endoscopy-assisted ACDF surgery

This surgical technique is based on the standard ACDF surgical procedure, assisted by endoscopy for spinal canal decompression. This technique can provide a clearer surgical field, increase the tissue discrimination, and improve safety. In addition, the application of the rigid bendable high-speed endoscopic burr provides a broader range of decompression. These are conducive to achieve complete decompression. All patients underwent this surgical technique performed by the same senior surgeon.

**Figure 1:**
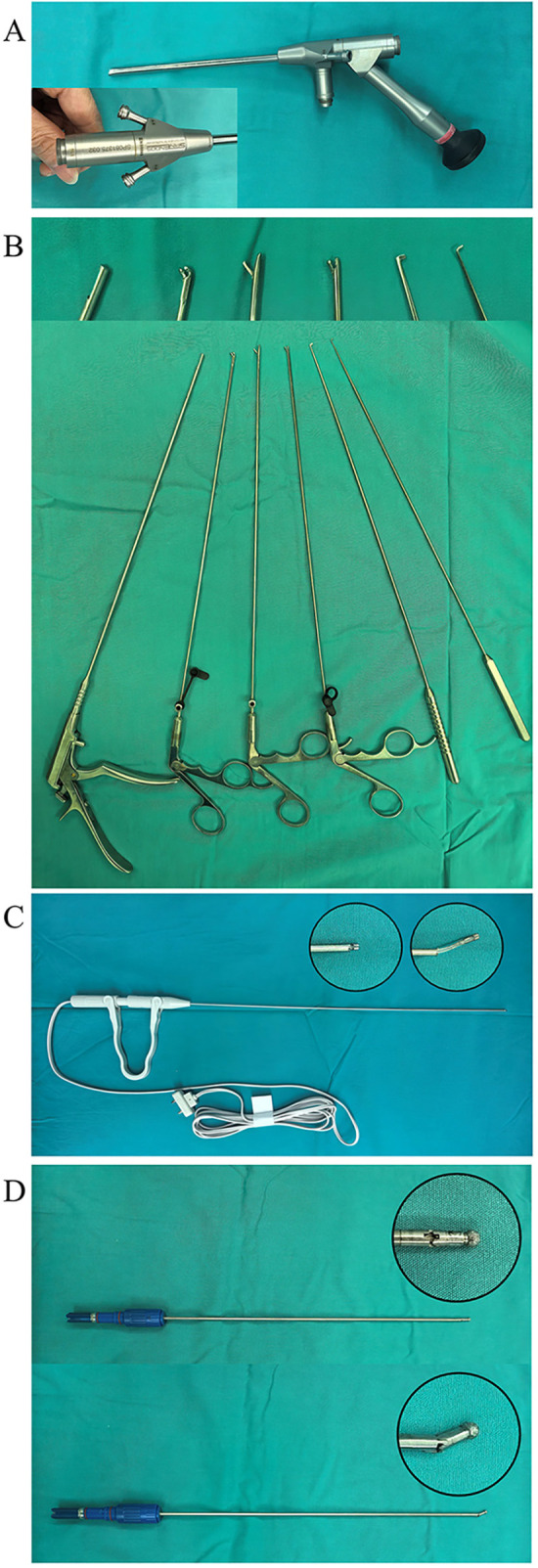
The spinal endoscopy system comprising **(A)** coaxial spinal endoscope, **(B)** coaxial spinal endoscopic instrumentsta, **(C)** bipolar radiofrequency electrode and **(D)** a disposable sterile articulating high-speed burr.

Under general anesthesia, the patient was placed in supine position. Natural cervical extension was achieved by positioning thin surgical cushions beneath the neck, shoulders and back. Preoperatively, the awake patient had been assessed by simulating the intraoperative cervical extension position to evaluate potential spinal cord dysfunction aggravation and prevent iatrogenic neurological deterioration secondary to mal-positioning. Bilateral upper limbs were maintained in distal traction and securely fixed with adhesive tape. The target level was identified under C-arm fluoroscopy guidance, and the incision site was marked. Routine skin antisepsis and draping, with the addition of waterproof surgical drapes with integrated drainage pouches. The spinal endoscopy system was prepared for deployment, comprising (Figure 1): coaxial spinal endoscope, coaxial spinal endoscopic instrumentsta, bipolar radiofrequency electrode (DTF-40, Elliquence Trigger-Flex®Bipolar System, Elliquence, USA) and a disposable sterile articulating high-speed burr (LB29035J.DS, Chongqing Xishan Science & Technology Co., Ltd., China).

The standard ACDF procedure was followed until the target disc was removed. A 2 cm transverse incision was made on the right side of the neck. The skin, subcutaneous tissue and platysma were cut in turn. The deep cervical fascia was exposed by blunt separation, along the deep layer of the platysma in cephalad and caudad directions. After the deep fascia was longitudinally incised along the medial border of the sternocleidomastoid muscle, the potential loose connective tissue between the visceral and vascular sheaths was bluntly separated to reach the anterior vertebral body. The prevertebral fascia was exposed and incised, and the target disc was fully visualized, which needs to be re-confirmed by C-arm fluoroscopy. The surgical field was fully exposed, with cephalad and caudad partial vertebral bodies adjacent to the target disc and bilateral medial borders of the longus colli muscles. The Caspar retractor was positioned to maintain adequate exposure. Under direct visualization, the annulus fibrosus (AF) was sharply incised with a #11 scalpel, and the nucleus pulposus (NP) was meticulously removed with curettes and pituitary rongeurs. When the posterior vertebral body and bilateral Luschka joints were exposed within the intervertebral space, it was the turn of the spine endoscopy.

**Figure 2:**
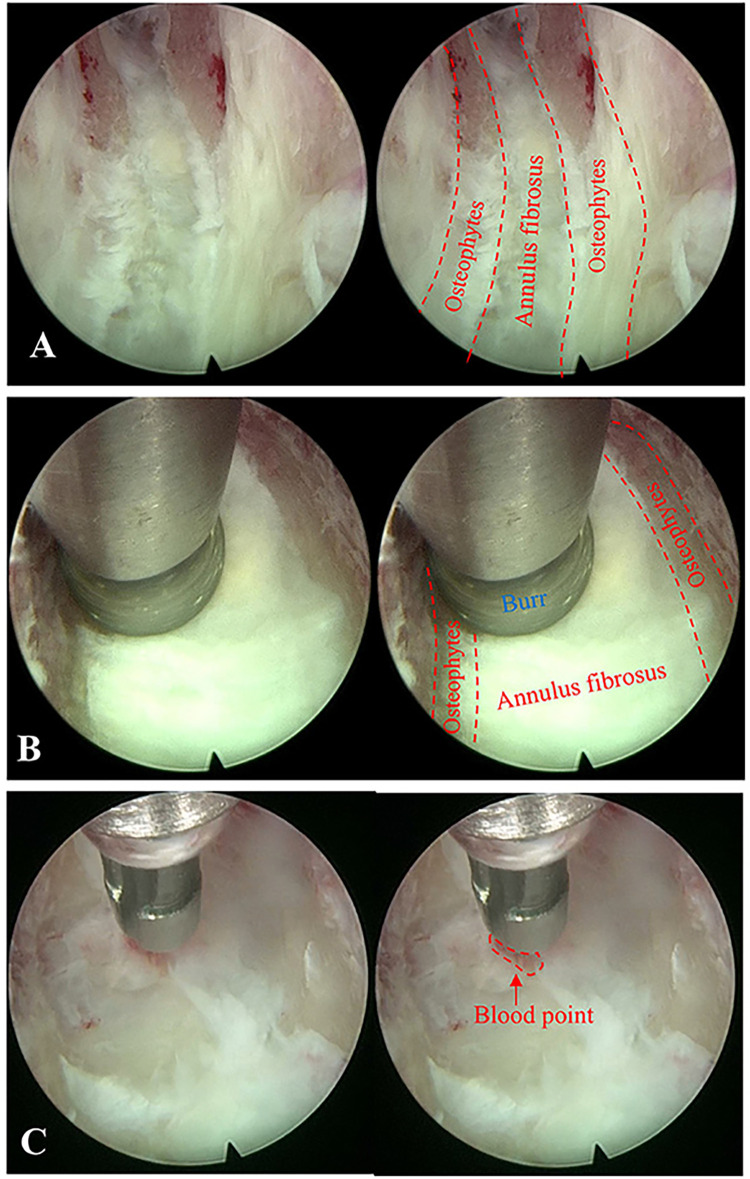
Field of views under endoscope during endoscopy-assisted anterior cervical discectomy and fusion (ACDF) surgery. **(A)** The target disc and posterior vertebral osteophytes were recognized under endoscope. **(B)** The burr was used to remove posterior vertebral osteophytes and the attachment sites of the annulus fibrosus. **(C)** Bipolar radiofrequency was used to achieve hemostasis.

**Figure 3:**
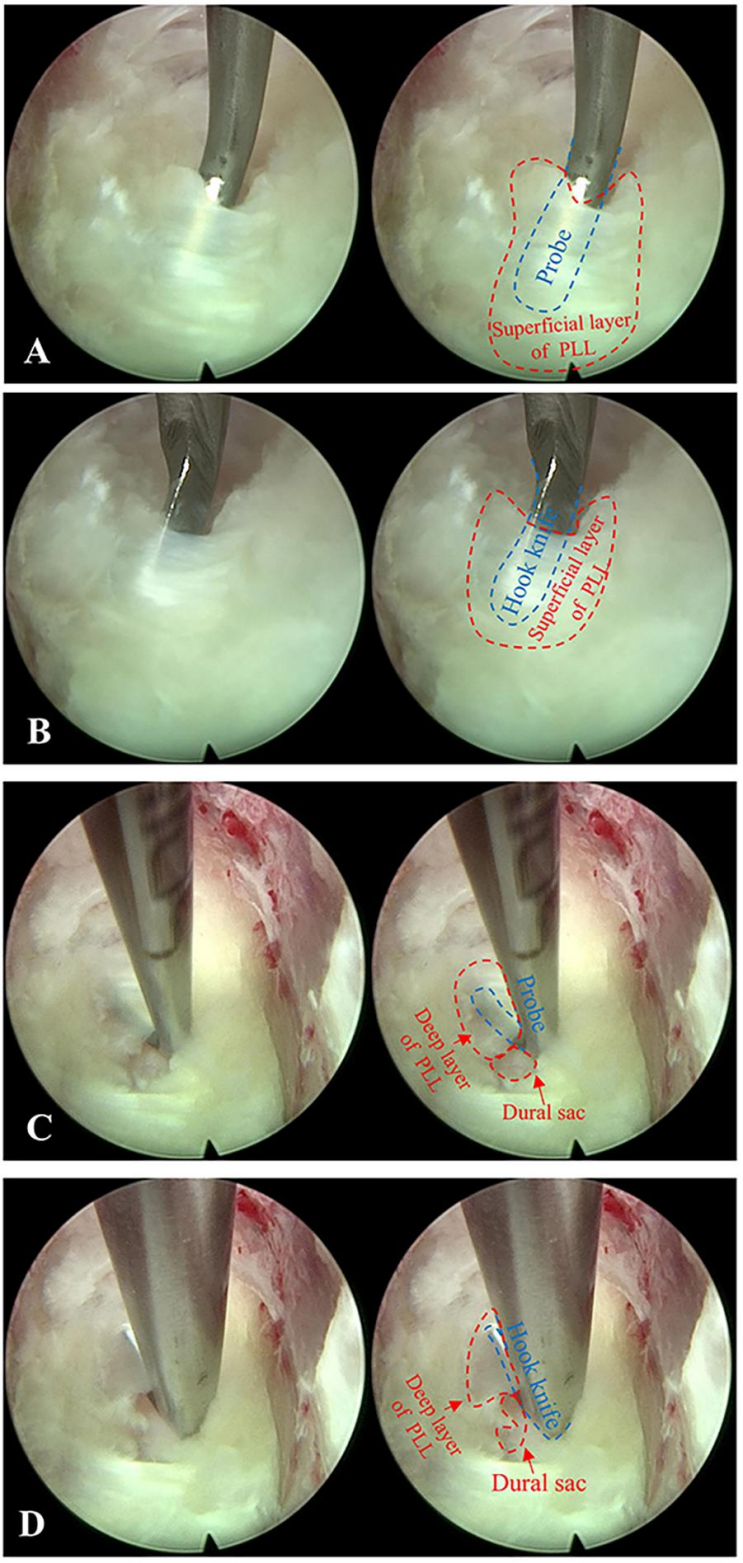
Removal of the posterior longitudinal ligament (PLL) under endoscope. **(A)** A probe was used to dissect between superficial and deep layers of PLL. **(B)** A hook knife was used to open the superficial layer of PLL. **(C)** A probe was used to dissect between the deep layer of PLL and the dural sac. **(D)** A hook knife was used to open the deep layer of PLL.

**Figure 4:**
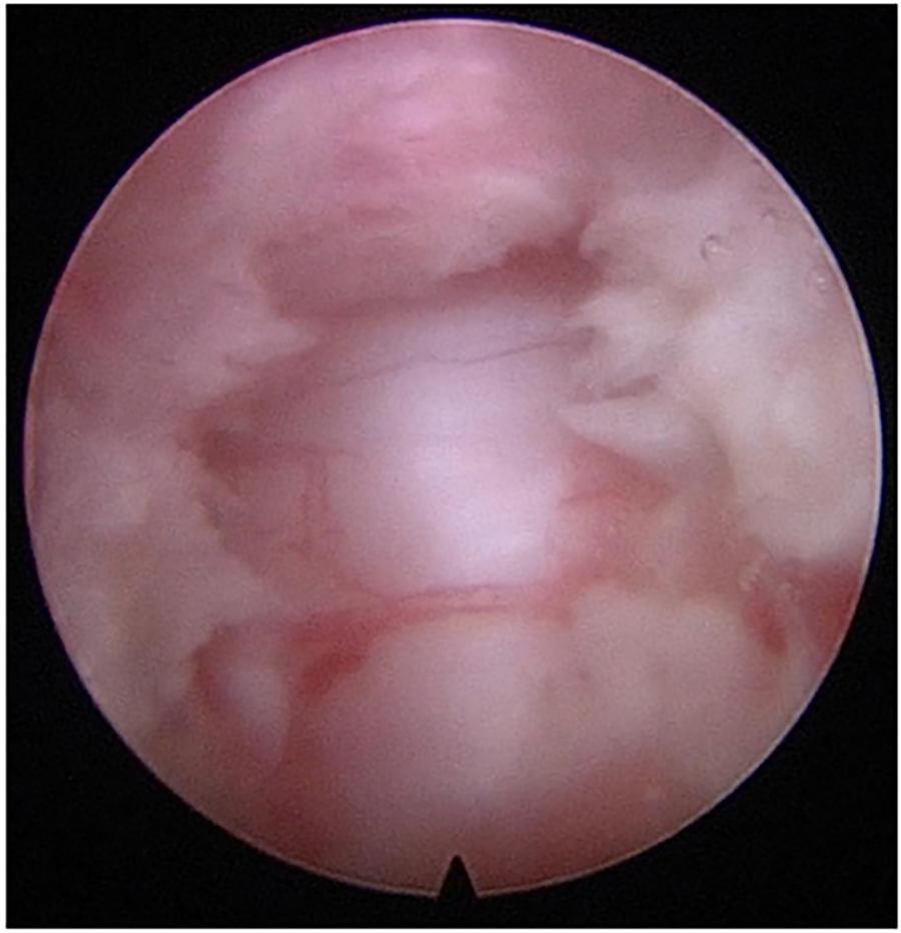
The dural sac was exposed fully under endoscope.

Notably, the application of spinal endoscopy is the key to the surgical process. The uniaxial spinal endoscope was inserted into the intervertebral space with appropriate space distraction, and the surgical field was transitioned from direct eyesight to endoscopic visualization with fluid medium. Continuous irrigation flow during endoscopic operation is essential to ensure optimal visualization clarity. The endoscope was advanced incrementally with real-time modulation of intervertebral space distraction to optimize surgical exposure. Simultaneously, adjustment of the 30 ° field of view enhanced observation and removal of posterior vertebral osteophytes and migrated disc fragments (Figure 2A). Under monitoring of endoscopic, the rigid bendable high-speed endoscopic burr was utilized to meticulously remove posterior vertebral osteophytes and the attachment sites of the annulus fibrosus (Figure 2B), and intermittent application of bipolar radiofrequency was required to achieve hemostasis and prophylactic coagulation to support visualization clarity (Figure 2C). Residual NP and AF, remaining from direct visualization operation, were removed with endoscopic pituitary rongeurs. The posterior longitudinal ligament (PLL) was exposed, and a probe was used to dissect between its superficial and deep layers (Figure 3A), followed by incision of the superficial layer with a hook knife (Figure 3B). Similarly, the deep layer of PLL and the dural sac were separated by a probe (Figure 3C), and the deep layer of PLL was opened by a hook knife (Figure 3D). Basket forceps or thin laminectomy rongeurs were then used to continue resection of the AF and PLL, achieving full exposure of the dural sac (Figure 4). Finally, the nerve dissector was utilized to thoroughly explore cephalad and caudad directions for residual NP tissue to ensure adequate decompression. The burr was used to gently abrade the remaining superior and inferior cartilaginous endplates, and bipolar radiofrequency and flowable gelatin hemostatic matrix were applied to achieve hemostasis. Once active bleeding was confirmed absent, the spinal endoscope was gradually withdrawn.

Following confirmation of satisfactory decompression, a cage with allogeneic bone graft was inserted into the disc space and secured with a plate-and-screw construct, and the satisfactory positioning of these implants was confirmed by C-arm fluoroscopy. The wound was irrigated with normal saline, drainage was placed, and the incision was sutured layer by layer and bandaged with sterile dressings.

### 2.3 Postoperative management

Cephalosporin was routinely used to prevent infection within 24 h after operation. The drainage tube was removed according to drainage volume on the first day after operation, and postoperative x-ray, CT and MRI were reviewed. The patients were asked to wear a neck brace during ambulation for the first 2 weeks postoperatively. And they were informed of regular outpatient follow-up.

### 2.4 Clinical assessment

Operative time and postoperative drainage volume were recorded. Complications were recorded, including dyspnea, hoarseness, dysphagia with aspiration, cerebrospinal fluid (CSF) leakage, nerve injury, and hematoma. Japanese Orthopedic Association (JOA) score, a scale used to assess neurological function, was evaluated preoperatively and at the last follow-up. The improvement rate of JOA score was calculated as (last follow-up JOA score—preoperative JOA score)/(17—preoperative JOA score) × 100%, and categorized as excellent (75%∼100%), good (50%∼75%), fair (25%∼50%), or poor (<25%).

### 2.5 Imageological assessment

Lateral x-ray was used to evaluate the cervical curvature, and the change between preoperative and postoperative curvature was calculated. C2-C7 Cobb angle was measured, which was defined as angle formed between the inferior endplates of the C2 and C7 vertebral bodies. *Δ* Cobb angle was calculated as (postoperative minus preoperative C2-C7 Cobb angle). CT was used to evaluate the osteophyte removal and interbody fusion. Interbody fusion was defined as that continuous trabecular bone was observed between adjacent vertebral bodies at the last follow-up (3). MRI was used to evaluate decompression of the spinal cord.

### 2.6 Statistical analysis

Data analysis was done using IBM SPSS Statistics (version 25.0; IBM Corp., Armonk, N.Y., USA). Continuous variables were presented as mean ± SD. In order to compare the difference between pre-operation and the last follow-up, paired t test was used for analysis. A two-sided *P* < 0.05 was considered statistically significant.

## 3 Results

### 3.1 Clinical assessment

All 35 patients underwent endoscopy-assisted ACDF surgery successfully, and were followed up for 12.9 ± 2.1 months (range 9∼18 months). The operation time was 74.4 ± 10.7 min (range 60∼100 min). Postoperative drainage volume was 14.1 ± 5.8 mL (range 5∼25 mL). No complications were observed, including dyspnea, hoarseness, dysphagia with aspiration, CSF leakage, nerve injury, or hematoma.

There was no aggravation of neurological symptoms after operation, and the JOA score at the last follow-up was significantly improved compared with that before operation (15.7 ± 0.8 vs. 10.3 ± 1.9, *P* < 0.001). At the last follow-up, according to the improvement rate of JOA score, 25 patients were considered excellent, and 10 patients were considered good. No fair or poor was observed. The excellent and good rate was 100% (35/35).

### 3.2 Imageological assessment

At the last follow-up, the C2-C7 Cobb angle was significantly higher than that before operation (26.3˚±4.3˚ vs. 18.9˚±4.6˚, *P* < 0.001), and *Δ* Cobb angle was 7.4˚±2.5˚ (range 3˚∼17˚). The osteophyte removal was satisfactory, which was evaluated on CT and MRI. All patients achieved interbody fusion, and the fusion rate was 100% (35/35).

### 3.3 Typical case report

A 71-year-old female complained of numbness in both hands associated with gait disturbance for 6 months, and the symptoms was worse in the past week. On admission, she presented with numbness in both hands and gait disturbance with a sensation of “walking on cotton wool”.

**Figure 5:**
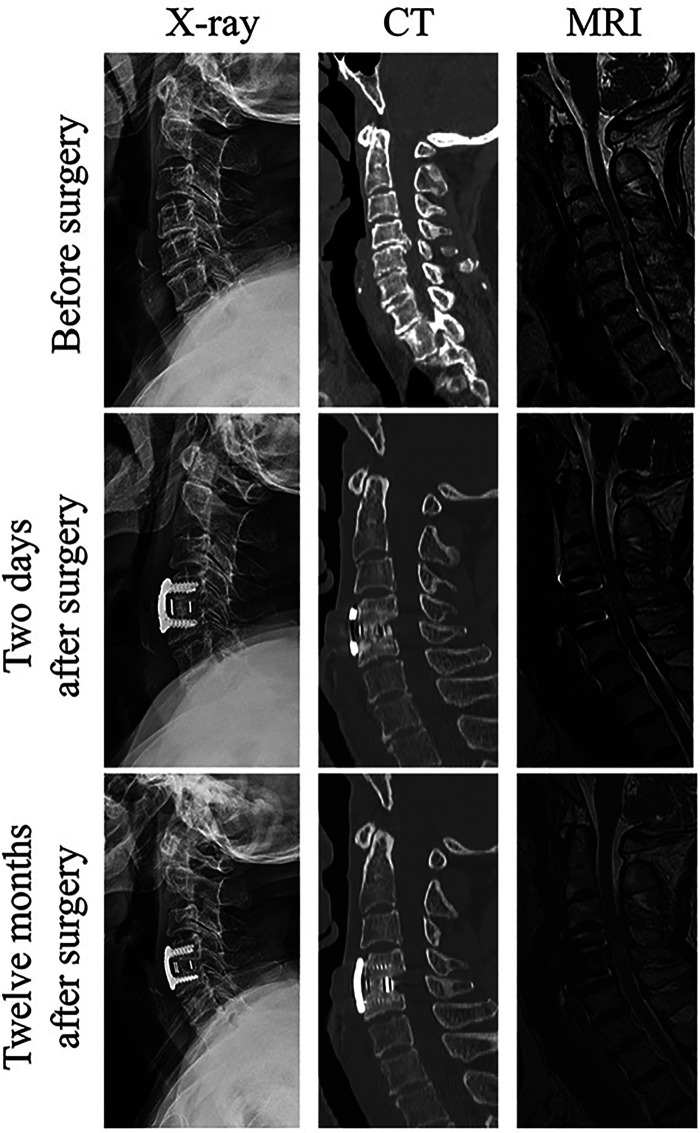
The imageological examination of a 71-year-old female typical case before and after surgery and at the last follow-up. Continuous trabecular bone crossing the cage was observed in the mid-sagittal CT view at the 12-month follow-up, confirming interbody fusion.

Physical examination revealed tenderness and percussion pain over the cervical and upper thoracic spine with bilaterally positive Hoffmann's signs and Babinski's signs. Neck extension induced numbness in the right upper limb. For imageological examination, cervical MRI showed the C4-5 disc herniation compressing the dural sac, and cervical CT showed posterior vertebral osteophytes and calcification of the C4-5 disc (Figure 5).

The patient was diagnosed with CSM, and successfully underwent endoscopy-assisted ACDF surgery at C4-5 level with an operation time of 90 min. Postoperatively, her symptoms improved significantly. The JOA score was from 12 preoperatively to 17 at the 12-month follow-up, and the C2-C7 Cobb angle was from 15 ° preoperatively to 21 ° at the 12-month follow-up, indicating neurological function recovery and of cervical lordosis restoration. In the mid-sagittal CT view at the 12-month follow-up, continuous trabecular bone crossing the cage was observed between C4 and C5 vertebral bodies, confirming interbody fusion (Figure 5).

## 4 Discussion

Cervical spondylotic myelopathy (CSM) is one of the most common causes of chronic spinal cord injury in adults, resulting in decreased hand dexterity and gait instability, as well as sensory and motor dysfunction (4, 5). Therefore, once identified, CSM usually requires surgical intervention (6, 7). In the past, traditional open ACDF has been widely accepted by spine surgeons. However, this procedure has the disadvantages of narrow surgical field, inadequate illumination, and challenges in achieving hemostasis. Especially for patients with ossification of the posterior longitudinal ligament or distant extrusion of the nucleus pulposus, this procedure usually can not completely remove the compressors, and is associated with the elevated risk of intraoperative spinal cord injury.

In recent years, microscopy-assisted ACDF has entered the sight of spinal surgeons and achieved satisfactory clinical outcomes in the treatment of CSM (8, 9). However, the major limitation of microscopy-assisted ACDF was the fixed positioning of the surgical field and microscope lens, which precluded simultaneous focus on multiple surgical planes. When extensive manipulation within the intervertebral space was required, or when any minor collision or deviation leaded to loss of the surgical field, the microscope must be recalibated and refocused, thereby prolonging operative time and increases the potential risk of surgical infections due to intraoperative contamination (10).

Percutaneous spinal endoscopic techniques have been widely adopted in the surgical treatment of degenerative spinal diseases across the entire spine, including the cervical, thoracic, and lumbar regions, demonstrating favorable clinical outcomes (11–13). The combination of uniaxial endoscopy and traditional ACDF, i.e., endoscopy-assisted ACDF, shows significant advantages (14, 15). (1) Enhanced surgical visualization. The spinal endoscope, equipped with an integrated light source, can accurately find and timely stanch the bleeding point under the fluid medium. The intraoperative blood loss cannot be recorded due to it was irrigated away by the fluid medium. So, we recorded the postoperative drainage volume. The low drainage volume, averaging 14.1 mL, was closely attributed to meticulous hemostasis under a clear visual field. (2) Superior tissue discrimination. Under endoscopic visualization, the layered longitudinal PLL can be clearly differentiated from the dural sac. During tissue separation and resection, the target tissue can be completely removed while avoiding damage to nerve roots, spinal cord, and dural sac. In this study, there were no complications such as neurological deterioration, which depended on the intraoperative clear exposure and complete decompression. (3) Expanded visualization and operation area. The enhanced surgical accessibility included three critical aspects: extended visualization via a 30 ° field of view spinal endoscope, augmented bone resection region via a rigid bendable high-speed endoscopic burr, and expanded hemostasis range via a bipolar radiofrequency electrode with flexible head. These instrumentations synergistically allowed for safe and complete decompression of the posterior vertebral edge, intervertebral space and Luschka joints, and achieved V-shaped decompression of the posterior vertebral edges and release of nerve roots. Notably, while intraoperative hemorrhage remains a persistent challenge in endoscopic spine techniques (16), our protocol successfully mitigated this limitation through the coordinated application of these instrumentations. The burr's dynamic angulation combined with the electrode's articulating range permitted efficient hemostasis across the expanded surgical field. In this study, the JOA score after surgery was significantly improved compared with that before surgery, depending on the complete and adequate decompression of the spinal cord. (4) Coaxial visualization and operation. Tang et al. proposed that the mutual occlusion between endoscopes and instruments makes it relatively difficult to operate large Kerrison punch and pituitary forceps (16). However, coaxial spinal endoscopy eliminated visual obstruction caused by operating instruments and reduce reliance on assistant coordination, and also avoided the defect of “hand-eye dissociation” of microscopy. Furthermore, the operation process was displayed and recorded in real-time on a monitor, which was conducive to the learning, training and knowledge exchange among junior surgeons. The limitations of this technique include the steep learning curve and the difficulty in promptly detecting CSF leakage under the fluid medium.

**Figure 6:**
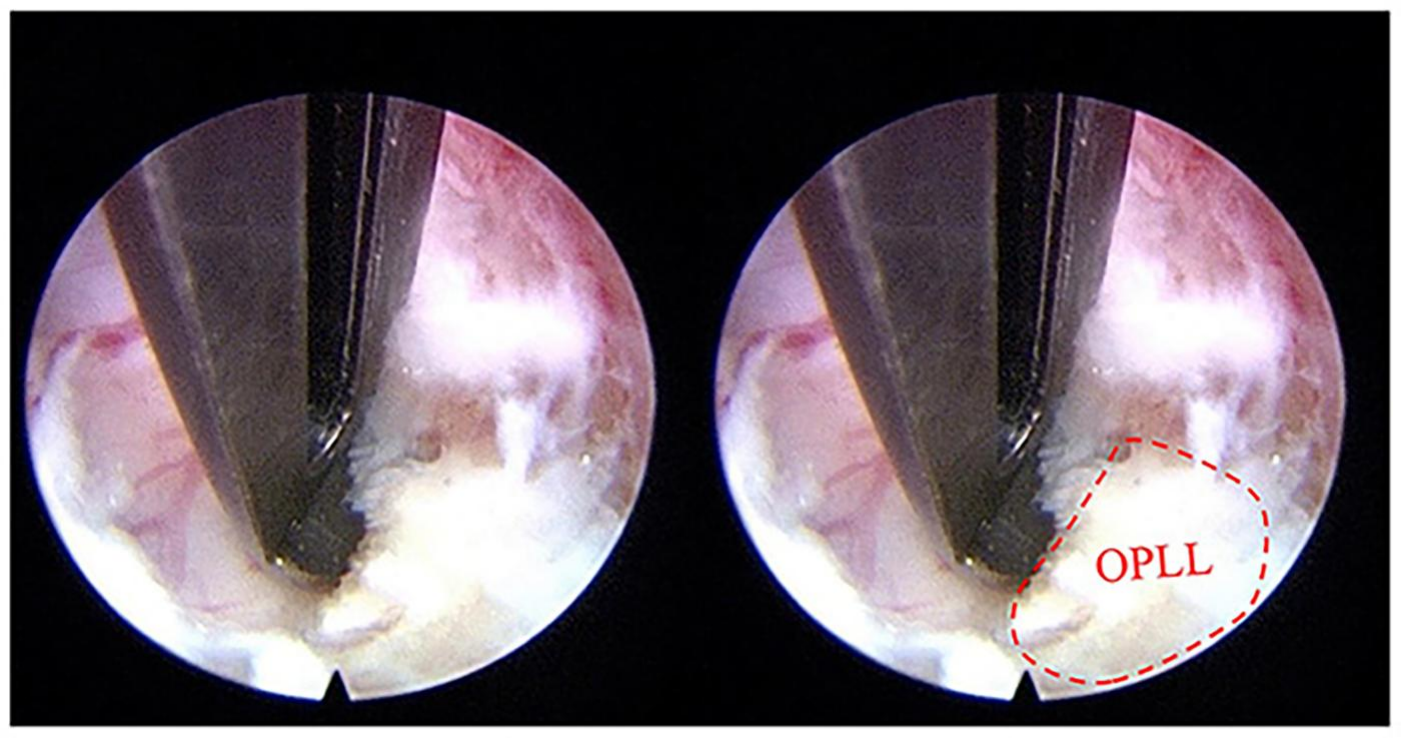
Ossification of posterior longitudinal ligament (OPLL) was removed under endoscope.

To ensure successful completion of the procedure, there are some intraoperative key points that need to be emphasized. (1) Maintaining endoscopic stability. Avoid over-distraction of the intervertebral space and keep the endoscope stuck in the space to stabilize the lens, which can maintain a stable field of vision and relieve muscle fatigue of the endoscope-holding hand. When the posterior vertebral edge needed to be treated, adjust the Casper by appropriate distraction to allow the endoscope advance deeper. (2) Appropriate osteophyte resection. Prioritize side-grinding and pull-up grinding techniques to avoid excessive removal of superficial bone, so as to protect the bony endplate. The 30 ° endoscopy with its swing tilt and the rigid bendable high-speed endoscopic burr can further expand the decompression range effectively. (3) Meticulous dural sac exposure. A probe was used to carefully separate the space between the PLL and the dural sac, and then the basket forceps and hook knife were used to incise the PLL to fully expose the dural sac (Figure 4). In some cases with ossification of PLL (OPLL), the separation between the PLL and dural sac should be initiated at non-ossified regions of PLL. The probe was used to delicately detect whether there were adhesions between the ossification and the dural sac. Following complete separation, the ossification was progressively removed using 45 ° pituitary forceps or laminectomy rongeurs (Figure 6) until the dural sac was fully decompressed, and confirmed by satisfactory dural re-expansion and pulsation under endoscopic visualization.

There were 35 patients with CSM who underwent uniaxial endoscopy-assisted ACDF in this study. After more than 9 months of follow-up, significant improvements were observed in JOA scores and cervical lordosis and no postoperative complications occurred, including dyspnea, hoarseness, dysphagia with aspiration, CSF leakage, nerve injury, or hematoma. At the last follow-up, there was no internal fixation failure, and the fusion rate was 100%, which was attributed to complete abrasion of residual cartilaginous endplates with burr and effective protection of bony endplates under the endoscopic visualization.

The present study had several limitations. First, this study is retrospective and single-centered with a relatively small sample size, and the results need to be further validated by prospective, large sample, and multi-center studies. Second, only patients undergoing endoscopy-assisted ACDF were included in this study, and the absence of a control group (e.g., traditional ACDF or microscopy-assisted ACDF) limits the ability to draw comparative conclusions regarding the true advantages of endoscopic assistance. Third, the mean follow-up period of 12.9 months only demonstrates satisfactory short-term outcomes. However, bony fusion and adjacent segment degeneration are time-dependent processes, and the longer-term results are still required to confirm durability and safety through extended follow-up periods.

## 5 Conclusion

Endoscopy-assisted ACDF, which combined the uniaxial spinal endoscopy with traditional ACDF, achieved satisfactory short-term clinical efficacy and safety in the treatment of CSM.

## Data Availability

The raw data supporting the conclusions of this article will be made available by the authors, without undue reservation.
